# Learning to see clearly again: How critical media engagement can save public health

**DOI:** 10.3389/fpubh.2026.1731417

**Published:** 2026-02-09

**Authors:** Sobi Thomas, Angel Maria Mathew

**Affiliations:** 1 Department of Communication and Media Studies, Marian College Kuttikkanam Autonomous, Kuttikkanam, India; 2 School of Public Health, Torrens University Australia, Adelaide, SA, Australia

**Keywords:** critical media engagement, digital media, digital resilience, education, health literacy, misinformation, public health communication

## Introduction

Trusted parties like healthcare professionals, government, and the media of proven history have been traditionally used to help in the dissemination of valid health information in the field of public health. This is, however, changing with the emergence of digital platforms (1). The online influencers, viral content, and unverifiable information now dominate health narratives to the masses and usually prevent authoritative sources of information. This change has resulted in a paradox, namely that the increased access to information has grown exponentially, but its quality and reliability have become less clear (2). The rise of the digital world has given rise to information overload phenomena, such that many people are often overwhelmed with all the information they receive, incapacitating them from knowing what they can and cannot rely on. This is the situation that has led to the creation of great demand for Critical Media Engagement (CME)—the new media literacy (3). CME can enable individuals' ability to critically interpret and comprehend health-related media content, that can empower them to navigate the aspects of the information ecosystem and make informed choices in an age of misinformation (4).

## The great irony: more information, less clarity

The digital era has created a paradox: on the one hand, the knowledge process is more democratic on social media, as much of the information is much more accessible to all, and on the other hand, it is easy to spread fake information. The viral posts and the sensationalized stories have been competing with the previously heavily edited health-related content, where the main priority of the algorithm is not the accuracy but the engagement (5). Emotions and fear in particular, play a significant role in the phenomenon since emotional contents tend to spread quickly than facts. The dynamics have resulted in the fact that the number of those who lost confidence in scientific institutions and health advice provided by the government has increased, and people are being inundated with various health claims in multiple channels (6). It is also quite discouraging because individuals tend to get puzzled and distrustful of all this conflicting information, making them mistrustful of the recommendations given by the experts. The new requirement on the edge of suspicion is the Critical Media Engagement (CME). CME might assist an individual to critically analyze health data, discover emotional manipulation, differentiate facts supported by evidence and false narratives, and make informed choices in the rapidly evolving digital environment where information is the order of the day (7). Promotional activities around CME should also address structural obstacles. In our view, political polarization, declining confidence in institutions, and ideologically segmented media environments can significantly complicate engagement with evidence-based health information. From this perspective, misinformation often aligns with identity and belief systems, making corrective communication particularly difficult in such contexts.

## What it means to engage critically

Critical Media Engagement (CME) is one of the most important digital skills in relation to the subject of public health literacy. Alongside related competencies such as digital health literacy, information evaluation skills, and online risk awareness, CME uniquely integrates critical analysis, contextual understanding, and ethical responsibility into health-related media use. It not only entails having the knowledge of health, but putting information in the media through analysis, questioning, and interpretation to make healthy and sound decisions (8). Through CME, people comprehend what they read in a superior way because they use messages on health, not as passive receivers of health information, but as active participants in the health information they read. Three core competencies define CME and comprise such aspects as Analytical Competence, which presupposes an evidence analysis, biases, and the capacity to draw the line between credible and invalid sources (9).

The Competence of the contextual aspect explains contextual knowledge about the impacts of cultural, social, and technological features on health messages and how they should be perceived. Finally, Ethical Competence enhances responsible journalism, such as circulating verified news or not encouraging the amplification of false news (10). These competencies have been combined to offer citizens the instruments they require to swim in the intricate media world, resulting in a more educated and skeptical population. Through the development of CME, individuals will have the chance to examine the contents on their health in a far more accommodating manner since they will have the authority to make better-informed, healthy decisions. Although CME is gaining attention in public health and communication scholarship, empirical research on its implementation and effectiveness remains limited. Current studies primarily conceptualize CME rather than evaluate its outcomes in real-world settings (11). Future research should examine how CME interventions influence health behaviors across different populations, particularly in contexts marked by political polarization, institutional distrust, and algorithmic amplification of misinformation.

## The human heart of the matter

Emotions are relevant to the decisions humans make about their health, as well as their media consumption, which in most cases shape how people think of and act in relation to their health information. People are often motivated by fear, hope, and the urge to be reassured to find health content that appeals to their emotions instead of promptly and objectively addressing their concerns (12). This emotional bond demonstrates the relevance of empathy in health communication. Health communicators of the modern era should also understand that to establish trust, it is necessary to not only deliver the data but also to listen, comprehend, and respond to the emotions of the audience (13). Compassionate health communicators are able to connect with their respective audiences on a more personal level and recognize fears and uncertainties without necessarily dismissing them. This is a tactic that would establish trust and make people more open to evidence-based health suggestions. Conversely, a condescending and factual tone might not appeal to audiences especially when the audience has already formed an aversion.

## Educators as architects of digital citizenship

By encouraging the principles of critical media in the classroom, the contribution of educators can be imperative to form responsible digital citizens (14). With the help of a synthesis of health literacy and media literacy, teachers ought to be capable of educating students on the skills of providing a critical assessment of health-based information online (15). Such a combination strategy will inform students that they need to reason about the source from which they are getting information, and they need to consider whether it is true or not, and be ethical in what they are doing online. It assists them in distrusting the nature of health messages, propagating fake news, and understanding the more abstract social and cultural pressures that influence health messages. Adapted, real-world interventions that can be provided to teachers are coaching students through exercises that break viral false health information, encouraging students to work in groups by breaking a myth, and encouraging students to ruminate on a digital journal or in a group discussion (16). These activities allow the students not to act as passive consumers in their online worlds, but to participate. By adopting the strategy of making students think critically and ethically, educators will be in a position to produce a generation of accountable digital citizens with the ability to combat misinformation and make logical health decisions (17).

## Policy and systems: making CME the norm

Systemic change is required in order to integrate Critical Media Engagement (CME) into education and policy systems to ensure its ability to combat misinformation and encourage public health. By including the national curricula and health communication strategy, involving the CME will mean that people will come prepared with a skill set that will enable them to adopt a critical attitude to digital health information (18). Such nations as Finland have already introduced the concept of media literacy education at the early education stage and enabled students to cope with the intricacies of the digital content very young (1). Equally, Australia has illustrated the use of social listening technologies to promulgate false health narratives by tracking real-time misinformation (19). Such international strategies underline the institutional support of CME, which is a proactive one. To expound, CME should be a national health communication need and health workers trained by governments and health organizations to develop digital empathy and reduce misinformation. Considering the omnipresence of CME, it is expected that the societies will become more resilient to misinformation, thus, making the process of making informed decisions easier and increasing the degree of trust toward health-related advice (20).

To the public health practitioner, CME can help clarify the need to use empathetic and dialogic communication implementation strategies that respect emotional and cultural milieus. The policy makers in the field of public policy should anticipate the inclusion of CME in the national curricula and the general public health communication models. Teachers play a central role of integrating the concept of critical media analysis into a systematic part of the health education curriculum. In regard to the general population, CME fosters critical interaction with media, urges strict verification before the information is leaked, and develops ethical activity in the digital health conversation.

## The ethical tightrope

The programs of public health usually balance on a thin line between information and unintentionally influencing behaviors. It is ethically compelling to be cautious about influencers, behavioral nudges or health-based content created by artificial intelligence as the approaches can focus on the engagement, rather than the true understanding. According to recent systematic reviews, the implementation of artificial intelligence in the field of public health communication has the potential to advance existing health disparities through the application of biased data, the lack of transparency in the deterministic process of algorithm decisions, and inequitable access to digital technologies, which means the need to seek media engagement approaches that are both ethical and equity-oriented (21). These tactics could increase the reach, but they can weaken autonomy based on unfavorable practices of influencing decision-making without being openly communicated (5). Besides, the emergence of influencers who may recommend health tips without scientific evidence may deceive vulnerable groups. One of the ways in which the issues can be addressed as part of Critical Media Engagement (CME) is to ensure that the health messages are not only valid, but represented ethically. The element of cultural inclusivity also plays a significant role, meaning the messages have to be culturally significant and relatable to a number of communities, considering their values and beliefs, as well as experience (22). There is a check on alienation or lack of understanding through cultural and scientific definitions of health content, and this results in effective communication among all the segments of society. Among education, ethics, and participation in the process of raising awareness in the field of the health of society, among other factors, Saxony plays a vital role that can ensure the integrity of trust of the community and create informed judgment.

## Digital resilience: the new immunity

Digital resilience may be viewed as the ability to step back, reexamine, and investigate before reacting to digital information, especially in the case of misinformation. Just as in a world where fake news disseminates very quickly, digital resilience can be viewed as a significant defense mechanism, which takes the form of the ability of a person to critically analyze what they have read rather than take what they see at face value (23). It is concerning the individual responsibility, the development of cognitive capacity to know truth and falsehood, or social responsibility to involve other persons in spreading the correct information. The person is required to build a culture of checking, waiting, and reflection over gut reactions to build digital resilience. Also, the effort of digital resilience building is a collective endeavor, which relies on open institutions and benevolent societies. The media houses and health authorities should be concerned with transparency, whereby they are expected to put the right information in an open format and get rid of the misinformation once it is recorded. Until then, people ought to support each other in becoming digitally literate, and it is the duty of the wider community to deal with issues that can negatively affect the population because of the risks of misinformation (8).

## From information to transformation

Critical Media Engagement (CME) provides an ethical and analytical framework for digital health communication, guiding how individuals critically interpret information, engage empathetically with others, and use technology responsibly. A digitized world model is the CME, which is used to evaluate and engage health data in an ethical and thoughtful manner (7). The health policy of the future can neither be subversive nor supportive of any agenda that propagates the sources and occurrences of the population voices, but find ways of breeding smarter and conscious societies. Such strategies are not only aimed at increasing the level of health messaging, but also developing an informed citizenry, able to critically assess health information, ask probing questions and draw well-informed conclusions. The change requires that the communication process is focused on empathy since the gap can be less when the communicator is able to understand the emotional and cultural context of health information and therefore generate trust. The perception of empathy and critical thinking as a higher value will be the strategic factor ensuring that the sphere of public health will not be superficial in its work, and will start concentrating on the actual change. The application of technology should also be accountable and support informed decision-making and the wellbeing of a group, and not inspire misinformation (10).

## Conclusion

Critical media competence must be developed to navigate the increasingly complex digital health information environment. It is not only that gauging, doubting, and interacting with the content on the digital level has become more essential than ever before, but also that misinformation is spreading at an extremely high rate. Given the fact that there should be continuous learning, it is clear that one ought to be sufficiently trained on how to criticize the validity of health messages. Besides the ability to think, remaining emotionally stable, and adhering to moral principles are also significant in alleviating the experienced psychological and social consequences of misinformation. It cannot be upon the individuals but rather on educators and policymakers, and they should include Critical Media Engagement (CME) both in the curriculum and in the programs of public health. By implementing CME, we empower citizens and allow them to make informed decisions, build trust with health systems, and counter the effects of fake news. Media literacy is capable of spreading and facilitating collective action to secure the lives of people during the digital era.
